# A high sensitivity assay for the inflammatory marker C-Reactive protein employing acoustic biosensing

**DOI:** 10.1186/1477-3155-6-5

**Published:** 2008-04-29

**Authors:** Jeffrey D McBride, Matthew A Cooper

**Affiliations:** 1 Akubio Ltd., 181 Cambridge Science Park, Cambridge, CB4 0GJ, UK

## Abstract

C-Reactive Protein (CRP) is an acute phase reactant routinely used as a biomarker to assess either infection or inflammatory processes such as autoimmune diseases. CRP also has demonstrated utility as a predictive marker of future risk of cardiovascular disease. A new method of immunoassay for the detection of C-Reactive Protein has been developed using Resonant Acoustic Profiling™ (RAP™) with comparable sensitivity to a high sensitivity CRP ELISA (hsCRP) but with considerable time efficiency (12 minutes turnaround time to result). In one method, standard solutions of CRP (0 to 231 ng/mL) or diluted spiked horse serum sample are injected through two sensor channels of a RAP™ biosensor. One contains a surface with sheep antibody to CRP, the other a control surface containing purified Sheep IgG. At the end of a 5-minute injection the initial rate of change in resonant frequency was proportional to CRP concentration. The initial rates of a second sandwich step of anti-CRP binding were also proportional to the sample CRP concentration and provided a more sensitive method for quantification of CRP. The lower limit of detection for the direct assay and the homogenous sandwich assay were both 20 ng/mL whereas for the direct sandwich assay the lower limit was 3 ng/mL. In a step towards a rapid clinical assay, diluted horse blood spiked with human CRP was passed over one sensor channel whilst a reference standard solution at the borderline cardiovascular risk level was passed over the other. A semi-quantities ratio was thus obtained indicative of sample CRP status. Overall, the present study revealed that CRP concentrations in serum that might be expected in both normal and pathological conditions can be detected in a time-efficient, label-free immunoassay with RAP™ detection technology with determined CRP concentrations in close agreement with those determined using a commercially available high sensitivity ELISA.

## Background

Advances in the development of biochips, and microfluidic devices in particular, offer the potential to monitor clinically relevant biomarkers in serum or other biological samples with economy in terms of sample volume, reagents and assay time. Whilst these can be semi-automated for higher throughput applications, there is likely to be more impact in small devices for near-patient and point of care applications [1,2].

Acoustic biosensors allow label-free detection of biomolecules and analysis of binding events [3,4]. Detection is based on a quartz crystal resonator. The mass of captured analyte by an immobilised receptor molecule on the surface is proportional to the resonant frequency [5]. Today, acoustic sensors are generally based on quartz crystal resonators that are found in common personal electronic devices such as mobile phones, computers and televisions, with over a billion units mass-produced each year [6]. We have developed a novel acoustic detection technology, which we term Resonant Acoustic Profiling (RAP™; [6]). This technology builds on the fundamental basics of the "quartz crystal microbalance" or "QCM". Readout data is generated in real time, which can be analyzed to provide quantitative information including analyte concentration, analyte-receptor interaction specificities, affinities, and kinetics. In this paper we apply RAP to a clinically-relevant application, namely [CRP] estimation.

CRP is a classical acute phase reactant discovered by Tillett and Francis in the 1930s [7]. Although a fairly non-specific biomarker, the circulating concentration of CRP rises rapidly (within hours) in response to most forms of tissue damage, infection, and other acute inflammatory events including autoimmune diseases and malignancy. Since CRP can be elevated by as much as 1000-fold over baseline (~100 μg/L to as much as 500 mg/L), monitoring is considered very useful, not just for screening, but also for disease management since the level reflects not only the presence, but also intensity of inflammation or infection. Further, CRP is stable with a long plasma half-life (about 19 hours), remaining fairly constant with no diurnal or feeding induced variation [8]. In healthy blood donors, the median concentration is 0.8 μg/mL, the 90^th ^percentile is 3 μg/mL and the 99^th ^percentile is 10 μg/mL [8]. Routine commercially available assays for CRP quantification employ immunonephelometric and immunoturbidometric methods for CRP with ranges 3 to 8 μg/mL. Rapid tests have been developed for point of care CRP applications, particularly with reference to management of bacterial infections [9,10]. These tests are however of relatively low sensitivity with cut off values greater than 5 μg/mL.

Chronic inflammation is also an important component in the development of atherosclerosis. A number of studies have demonstrated the utility of CRP as a sensitive biomarker of cardiovascular diseases, in particular, future coronary heart disease (CHD), independent of traditional risk factors [11-16]. Thus, the assessment of CRP levels could provide a predictive method to assess cardiovascular risk, or assess the potential risk of recurrent cardiovascular events [17]. The association between CRP and CHD is similar to that of traditional lipid risk factors [16,18-20]. A cut off level for CRP of 2–3 μg/ml has been suggested [21,22]. The American Heart Association and the Centers for Disease Control and Prevention (AHA/CDC) clinically assessed a number of inflammatory markers [23]. CRP had characteristics considered most useful for practice, although mass screening at this stage was considered unwarranted. Their guidelines suggest that CRP measurement be taken twice over a two week interval, less than 1 μg/L CRP is 'low cardiovascular risk", 1 to 3 μg/mL is 'average' and greater than 3 μg/mL is 'high'. Values greater than 10 μg/mL should be repeated with the patient being examined for sources of inflammation or infection. Since this range includes levels in otherwise apparently healthy individuals, high-sensitivity CRP (hs-CRP) methods are required having limits of detection below that of routine assays (3 μg/mL). Automated immunonephelometric, immunoturbidometric methods now exist with assay ranges from as low as 50 ng/mL to 10 μg/mL and an immunoluminometric method has a range 100 ng/mL to 250 μg/mL for [24]. In addition commercial hs-CRP ELISA now exist with sensitivities as low as 1 to 5 ng/mL (American Diagnostica; Kalon Biological) but with a range to 100 ng/mL.

Clearly, such methods are either inefficient in terms of time or not easily transferable as point of care assays in a high sensitivity format, so there is potential for new high sensitivity, rapid methods. Ideally, such a test might cover the dynamic range expected for both routine and high sensitivity assays. In addition, insights into the association of CRP levels and other diseases are likely to require rapid assays of varying sensitivity or in novel matrices [25]. Herein we report our initial studies using acoustic biosensor technology for CRP quantification in diluted serum and whole blood.

## Results and Discussion

### Standard RAP assay design and features

**Figure 1:**
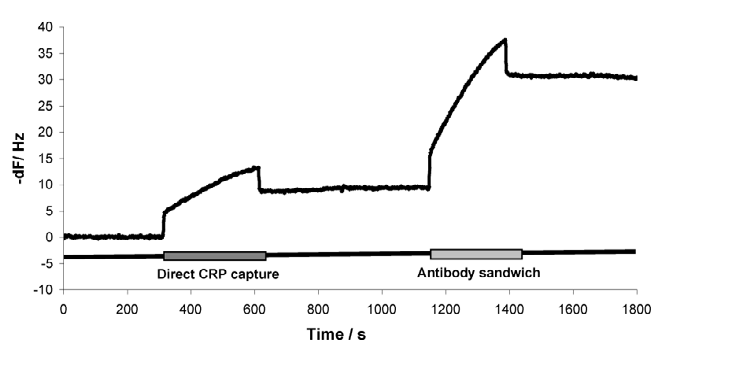
Real time analysis of [CRP] determination using RAP using a sandwich immunoassay. The trace shows typical data for the initial injection of a CRP containing sample (39 ng/mL CRP). Signal is seen as association of the CRP onto the capture antibody (Direct Capture Assay; t = 300–600 s). Next, sheep anti-CRP is injected to give a Direct Sandwich Assay. Again, an increase in signal is seen as association (t = 1100 – 1400 s).

The initial CRP assay carried out using RAP assay is designed around a two channel sensor 'chip'. Sheep anti-CRP was covalently coupled to the test channel using standard EDC/NHS coupling chemistry. Sheep IgG coupled to the other channel demonstrated very low background signal in the appropriately diluted spiked serum samples. Sample and/or standards are passed over these channels in parallel to give a fairly rapid assay of 30 minutes per cycle (Figure 1). CRP is often monitored in autoimmune diseases where samples containing rheumatoid factor have very high incidence [26]. The sheep IgG channel can provide a suitable control for both this or anti-animal antibodies that are a potential interference in immunoassays [27-30].

**Figure 2:**
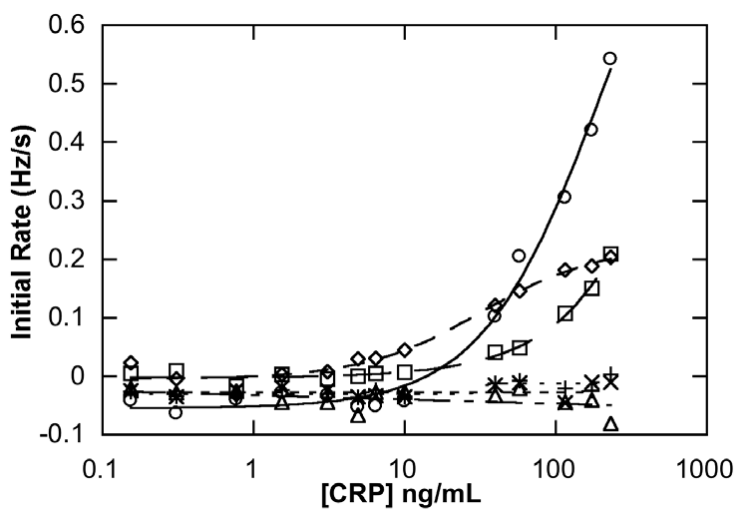
Three different CRP assay formats carried out using RAP showing both test channel (immobilised Sheep anti-CRP as capture antibody channels) and control channel (immobilised Sheep immunoglobulin G). Key: homogenous sandwich test channel (○); direct capture assay test channel (□); direct sandwich test channel (◇); homogenous sandwich control channel (x); direct capture control channel (+); direct sandwich control channel (Δ).

**Figure 3:**
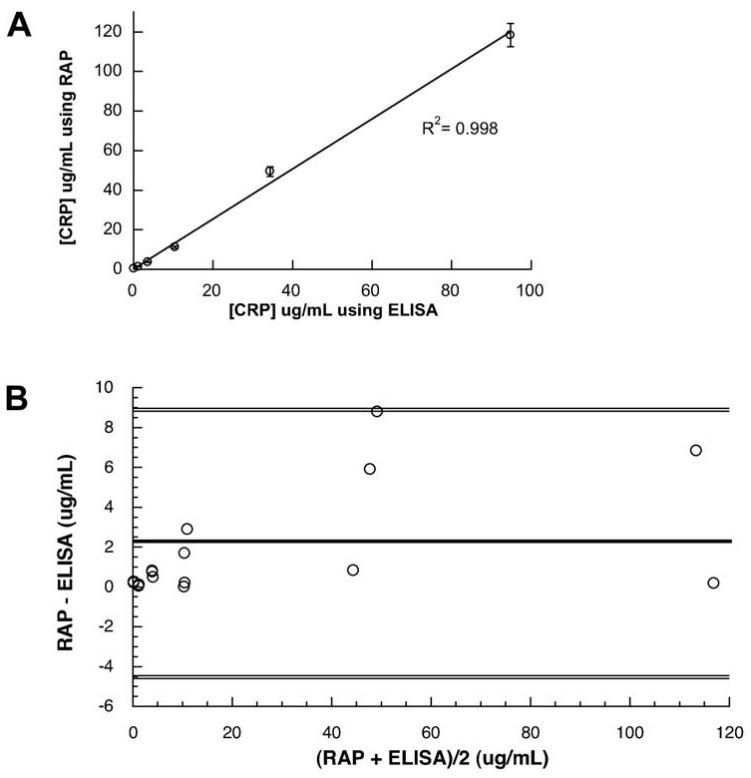
Comparison of CRP concentration found for spiked serum samples obtained by the direct sandwich CRP assay carried out using RAP with that of a commercial hsCRP ELISA a) Correlation plot, the x axis represents the values obtained by hsELISA, the y axis those values obtained by RAP b) Bland and Altman difference plot, the x axis represents the average of the RAP and ELISA values. The solid line is the mean value; dotted lines are 2 SD. The mean difference is 2.165 μg/L.

This initial CRP assay carried out using RAP assay was compared with a commercial, validated high sensitivity ELISA by analysing 6 spiked horse serum samples across the range of AHA/CDC guidelines. Good correlation for spiked serum samples above 5 μg/mL was found in all CRP assay formats using RAP (Figure 2) when compared to commercial hsCRP ELISA (Table 1). In particular, the direct sandwich assay also showed good correlation below this level (*R*^2 ^= 0.998; Table 1, Figure 3) The mean difference between the two methods for estimating serum CRP as calculated by Bland-Altman analysis (Figure 3b) was 2.17 μg/mL and the limits of the standard deviation (2SD) is 6.78 μg/mL. The differences in values obtained by the two methods lay within mean +/- 2SD. The methods agree well, whilst the RAP method gave higher values at 44 μg/mL and 116 μg/L, the difference at this level would not hinder classification according to the AHA/CDC guidelines and both methods would infer other sources of inflammation (bacterial, viral).

**Table 1:** Determination of serum CRP concentration using different RAP assay formats.

	**Cardiovascular Risk (AHA/CDC guidelines)/[CRP] g/mL**					
	LOW <1	Ave 1 – 3	HIGH 3 – 5	V. HIGH > 10		
	
Spiked Serum [CRP]	0.1	1.2	3.61	10.4	34.8	94.8
hsCRP ELISA	0.092	1.26	4.35	9.6	44	116
Direct Capture	3.728	4.53	7.56	11.9	41	113
Homogenous Sandwich	3.4	3.4	6.3	15.5	53	120
Direct Sandwich	0.14	1.45	3.3	9.9	45.5	114

Results are compared with that obtained by commercial hsCRP ELISA.

Cardiovascular risk is considered at serum levels > 3 μg/ml. Normal horse serum was independently spiked with human CRP, these samples were diluted from 1/100 to 1/6000 for assay. Sandwich assays using RAP employ 0.225 μg/mL Sheep anti-CRP.

The detection limit of the procedures was the amount of CRP that could produce a signal in the test (anti-CRP channel) equivalent to the mean value of duplicate zero mg/L CRP injections plus 3 times the standard deviation of the zero standard. For direct capture this was found to be 13 ng/mL, for homogenous sandwich Assay 20 ng/mL and for the direct Sandwich Assay 3 ng/mL.

**Figure 4:**
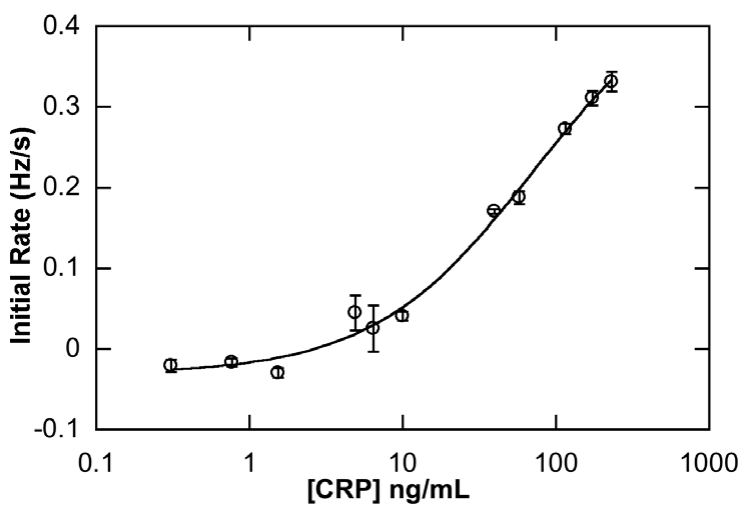
Direct Sandwich CRP assay carried out using RAP (n = 3).

Precision for the direct sandwich assay was determined using 3 test channels, injected with standard CRP concentrations from 0 to 232 ng/mL (Figure 4). Below 10 ng/mL the coefficient of variation (CV) rose above 10%, above this CRP concentration, a CV of 11.3% decreasing to 4.7% was observed.

An inter-assay, intra-assay precision profile analysis was performed by determining CRP concentrations of spiked serum samples using the sandwich assay in three to five replicates of each sample within test channels and different test channels (Table 2). The coefficient of variation (CV) lay between 3.1 to 12.6% across the range 0.1 to 116 mg/L original concentration of spiked serum. Generally acceptable CV values in diagnostic methods are less than 10%, the smaller the CV the more accurate the classification of sample. However, the level of imprecision found herein is similar to that of commercial hsELISA (e,g, IBL hsELISA, Hamburg, Germany quotes intra-assay CV of 5.5 and 6% for two samples of 22 and 99 ug/L CRP and inter-assay variation of 11.6 and 13.8% for two samples of 22.1 and 90.4 ug/L CRP) and the results still indicate the assay is useful in differentiating the cardiovascular risk levels.

**Table 2:** Precision analysis of CRP estimation by RAP.

**Sample**	**[CRP] g/mL Expected**	**[CRP] g/mL by hsELISA**	**[CRP] g/mL by RAP**	**RAP S.D**	**RAP %CV**
A	0.1	0.092	0.3 (n = 3)	0.026	8.82
B	1.2	1.26	1.3 (n = 3)	0.04	3.1
C	3.61	4.35	3.89 (n = 3)	0.45	11.6
D	10.4	9.6	11.34 (n = 4)	0.88	7.75
E	34.8	44	47.22 (n = 5)	5.97	12.65
F	94.8	116	116.9 (n = 4)	7.93	6.78

Horse sera spiked with human CRP and appropriately diluted was determined within and between runs using the direct sandwich format.

In order to test the possibility of false negative results due to high CRP levels, the standard CRP range was extended approximately two fold higher (231 ng/mL) than the hsELISA range. No hook effect was observed at this level (Figure 4).

### Rapid, semi-Quantitative RAP Assay

**Figure 5:**
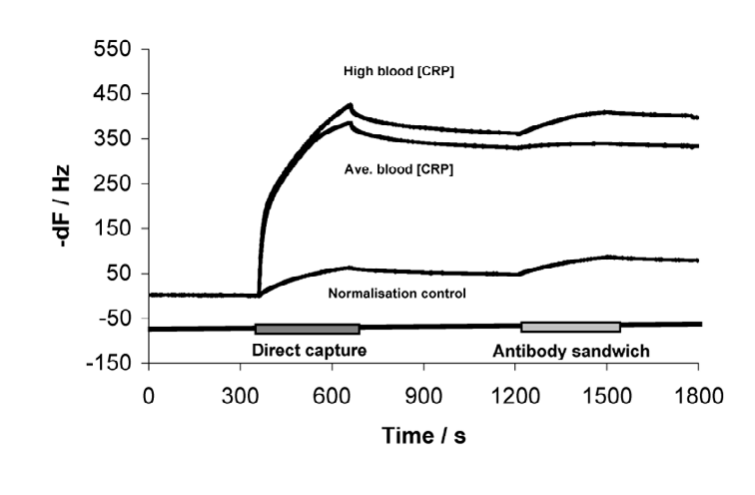
Sensorgram traces of individual test/control channels used for CRP in blood test; high level CRP blood, 6.818 μg/mL then diluted 1 in 50 in buffer (top trace); average level CRP blood, 0.68 μg/mL then diluted 1 in 50 in buffer (middle trace) and normalisation CRP standard of 27 ng/mL in buffer (equivalent to 3 μg/mL serum in whole blood diluted 1 in 50; bottom trace).

Whilst the standard CRP assay was able to provide quantitative results, calibration of a sensor channel response using standards prior to sample is a relatively time consuming process. Since the turnaround time is 10 minutes per sample, then five singlet calibration standards prior to a sample would take one hour turnaround. To deliver a more rapid semi-quantitative assay from a blood sample, a simple, rapid ratio metric assay was thus performed. A normalisation standard corresponding to a blood sample with 3 μg/mL CRP in serum was diluted 1 in 50 then passed over one channel. In parallel, a blood sample diluted 1 in 50 was passed over the other channel (Figure 5). The chosen CRP normalisation concentration corresponds to that of the 90^th ^percentile and also the borderline between 'average' and 'high' AHA/CDC guidelines. If the signal ratio between the two channels is greater than 1 then a higher CRP level is present and thus 'high' risk, if less than 1, 'low' risk and at 1 is borderline. Spiking of 3 separate blood samples at 'average' (1.5 μg/mL CRP in serum or 0.68 μg/mL in whole blood) and 3 separate blood samples at 'high' (15 μg/mL in serum or 68.18 μg/mL CRP in whole blood) CRP levels were tested. Ratios obtained gave excellent correlation with that expected for a calibrated sensor channel at these levels. For average CRP level blood the value obtained was 0.51 +/- 0.06 (expected ratio 0.5, n = 3) and for high CRP level blood the value was 1.45 +/- 0.2 (expected ratio 1.3, n = 3).

## Conclusion

CRP measurement as an indicator of inflammation or infection status is widely used. Assays for routine analysis are sensitive enough to determine from 5 μg/mL upwards since this had been considered the upper limit in the normal range [31]. Point of care assays have been developed for this range and are best suited to monitor clearly pathological conditions. The utility of serum CRP levels as a predictive test for CHD is now well documented and various hsCRP assays have been developed to monitor CRP levels within otherwise apparently healthy individuals. Such tests have included enzyme immunoassay and particle enhanced nephelometry and turbidometry [32,33], however these methods are either relatively time consuming or not directly suitable for adaptation into point of care methodology with high sensitivity.

The label free CRP assay carried out using RAP shows enormous potential in terms of both sensitivity and time efficiency. The protocol is amenable to both point of care and automation and in line with the range of CRP concentrations likely to be encountered. This includes concentrations above 5 μg/L traditionally monitored as an indicator of inflammation and/or infection, but also by virtue of its high sensitivity, those concentrations below this level, that span the guidelines recommended by AHA/CDC for cardiovascular risk. We note that the approach outlined in this paper could be extended to other markers associated with congestive heart failure found in blood and serum such as myoglobin, brain natriuretic peptide (BNP), NT-proBNP, and Troponin I (cTnI) to provide a comprehensive test panel for myocardial infarction, minor myocardial damage, and profiling of at risk and/or post operative patients with heart disease or a predisposition for heart disease.

## Methods

AKT◆*iv *sensor cassettes, 1-ethyl-3-[3-dimethylaminopropyl]carbodiimide hydrochloride (EDC), N-hydroxysuccinimide (NHS) (Akubio Ltd., UK), Dulbecco's modified phosphate buffered saline (PBS), Bovine Serum Albumin (BSA), Tris, Sodium Chloride, Tween-20, Sheep IgG were from Sigma-Aldrich (Poole, UK). Sheep anti-CRP, the hsCRP ELISA were from Kalon Biological (UK).

### hsELISA assay

Spiked horse serum was tested for CRP content using a validated commercial hsELISA kit from Kalon Biological (Kalon Biological, U.K.). The assay was conducted according to the manufacturer's instructions, with spiked serum diluted to as low as 1 in 5 to as much as 1 in 10,000 in the supplied sample diluent.

### Instrumentation and Sensors

RAP experiments were conducted using automated instruments (Akubio Ltd, Cambridge, UK). The instruments apply the principles of QCM, in that a high frequency voltage is applied to a piezo-electric crystal to induce the crystal to oscillate, and its resonance frequency is monitored in real time. The four-channel instruments comprise two pairs of oscillating crystal sensors mounted in parallel microfluidic flow cells, allowing sample to be flowed across four surfaces simultaneously. As sample is flowed across sensors, binding, if any, is measured as a reduction in the oscillation frequency.

The RAP instruments were fitted with a thermally-stable sensor mounting block providing temperature control, and with microfluidic and electrical connections to the piezo-electric sensors. Buffer flow was maintained with syringe pumps (Tecan UK Ltd, Reading, UK) under software control (Akubio Ltd., Cambridge, UK). Microfluidics comprised separate flow-paths to individual flow cells, combined with a common flow path split to address flow cells simultaneously. Interchange between the different flow paths was either by manual or electronically-operated valves (Akubio Ltd). Disposable AKT◆*iv *sensor covalent A acrylic sensor cassettes were employed in this work (Akubio Ltd., Cambridge, UK. These contain gold-coated quartz wafers with a carboxylic acid-terminated monolayer coating to provide a surface for protein immobilisation. Each cassette contains two derivatised sensors. These can be addressed independently via a micro-fluidic system that is integrated in to the AKT◆*iv *sensor cassette. One of the flow cells (channels) can be used as a control for real-time measurements if required. Two AKT◆*iv *sensor cassettes can be docked into Akubio's RAP instruments, allowing four simultaneous independent measurements to be carried out.

### Sensor Surface Preparation

Sensor surfaces were prepared by immobilising sheep anti-CRP onto the 'active' sensor surface and sheep immunoglobulin type G (Sh IgG) onto the 'control' sensor surface using conventional amine coupling chemistry. Immobilisation was performed at room temperature under continuous flow conditions with a running buffer, PBS, between sample injections was at a flow rate of 25 μL/min. Each injection step taking 3 minutes. First sensor surfaces were activated with a 1:1 mixture of 400 mmol/L EDC and 100 mmol/L NHS prepared in 0.22 μm-filtered deionised water, and mixed immediately prior to use (final concentrations; 200 mmol/L EDC and 50 mmol/L NHS). EDC-NHS was injected simultaneously across both sensor surfaces. Sheep anti-CRP and Sh IgG were prepared for immobilisation at 25 μg/mL in 10 mmol/L sodium acetate, pH 4.5, and were injected simultaneously across separate sensor surfaces. Non-reacted surface was then capped with BSA prepared at 100 μg/mL, again in 10 mM Sodium Acetate pH 4.5 and injected simultaneously across all sensor surfaces. Finally, the surface of sensors and microfluidic channels was blocked with 100 ug/mL BSA in Tris Buffered Saline (TBS). At the end of the procedure, between 412 Hz and 420 Hz of anti-CRP and 320 and 340 Hz Sheep IgG were immobilized on individual and approximately 630 Hz after capping/blocking with BSA on individual flow channels. The resulting "sensor chips" were stored at 4°C until required.

### Serum and Blood Sample Preperation

Normal horse serum spiked with human CRP was supplied by Kalon Biological (UK). Spiked horse blood was prepared as follows. Spiked whole horse blood collected in EDTA tubes was kept refrigerated and used within 24 hours of collection. The blood was centrifuged in 1.5 mL microcentrifuge tubes at 3000 × *g *for five minutes at 4°C, the upper layer was then aspirated. The whole blood volume was reconstituted by addition of the spiked serum appropriately diluted in normal serum to give blood spiked with human CRP at 1.5 μg/mL (average CRP) and 15 μg/mL (High).

### Standard RAP Assay for CRP

All assays were performed at room temperature under continuous flow at 25 μL/min with a running buffer of TBS, 0.005% Tween-20. CRP standards were prepared in a sample buffer comprising TBS containing 0.005% Tween-20 and 100 μg/mL BSA from a concentrated stock solution (94.8 μg/mL CRP). CRP spiked horse serum was also appropriately diluted in the same sample buffer from a 1/50 to 1/6000 dilution

#### Direct detection assay

CRP samples were prepared in sample buffer (TBS, 0.1% BSA, 0.005% Tween-20) These were injected for 5 minutes, and the initial rate of association was monitored.

#### Homogenous Sandwich Assay

Sheep Anti-CRP antibody (Kalon Biological, UK) was added to CRP containing standards and samples prior to injection to give a concentration of 0.225 μg/mL. The sample was then injected for 5 minutes.

#### Direct Sandwich Assay

Following the direct capture step above, anti-CRP antibody was injected for 5 minutes at a concentration of 0.225 μg/mL, and again the initial association was monitored.

The surface was regenerated after each assay by using a pulse of 100 mM Glycine-HCl pH 2.5 for 1 minute and re-equilibrated in TBST.

### Semi-quantitative RAP Assay for CRP

After gently mixing, spiked horse blood samples were diluted 1 in 50 in sample diluent (0.1 mg/mL BSA in 10 mM HEPES, 150 mM NaCl, 3 mM EDTA pH 7.4). The normalisation standard was 27 ng/mL CRP in the same buffer (equivalent to 3 μg/mL serum in whole blood diluted 1/50). A standard assay was then performed with Sheep anti-CRP sensor surfaces as previously but using running buffer of 10 mM HEPES, 150 mM NaCl, 3 mM EDTA pH 7.4. For each dual sensor channel chip, standard was passed over one channel, spiked sample over the other. The blood preparation and CRP screen was performed on three separate occasions. Blood samples were mixed by aspiration-dispense prior to loading onto sensor surface.

### Data Analysis Methods

RAP data was analysed as initial rates of signal generation (Hz/s) upon injection of CRP or antibody onto a test channel containing immobilised anti-CRP. The data was displayed and analyzed using RAP◆*id *Workbench v1.0.25 (Akubio Ltd., Cambridge, U.K.). Statistics were generated using Excel, and estimation of spiked samples was performed using a 4-parameter plot of the standards and appropriate dilution of the unknown spiked sera. For the semi-quantitative assay, the initial rate of signal at the sandwich step was corrected for baseline slope and the ratio of blood sample signal to normalisation standard signal was estimated.

## Competing interests

Certain commercial entities, equipment or materials are identified in this paper to describe the new assay. This is not intended to imply recommendation nor that the entities, material or equipment is best suited for the purpose.

Redundant publications: no substantial overlapping with previous papers.

## Authors' contributions

JDM designed and carried out the assay adaptation to acoustic biosensors. JDM and MAC wrote the manuscript.
